# Morphological changes of telocytes in camel efferent ductules in response to seasonal variations during the reproductive cycle

**DOI:** 10.1038/s41598-019-41143-y

**Published:** 2019-03-14

**Authors:** Fatma M. Abdel-Maksoud, Hanan H. Abd-Elhafeez, Soha A. Soliman

**Affiliations:** 1 Department of Anatomy and Histology, Faculty of Veterinary Medicine, Assiut University, Assiut, Egypt; 2 Department of Histology, Faculty of Veterinary Medicine, South Valley University, Qena, Egypt

## Abstract

Telocytes (TCs) are a distinct stromal cell type described in many organs. The present study investigated the existence of TCs within the efferent ductules in camel and the changes that occur in their morphology and activity during active and inactive reproductive seasons. TCs in the camel had a cell body and multiple telopodes (TPs), and most TCs had indented nuclei that exhibited prominent intranucleolar chromatin. TCs exhibited seasonal differences which were evaluated by histochemistry, immunohistochemistry (IHC), Transimition electron microscopy (TEM) and scanning electron microscopy (SEM). The presence of TCs in camel efferent ductules has been confirmed by CD34 positive immunostaing. In addition to the expression of the vascular endothelial growth factor (VEGF) which was stronger in the summer season. TCs exhibited stronger immunoreactivity for progesterone and oestrogen alpha receptors (ESR1) in the spring than in the summer. In addition, TCs showed strong positive immunostaining for both vimentin and androgen receptor (AR). Several ultrastructural changes were observed in TCs during the two seasons. TPs in the summer season had delicate ramifications whereas, in the spring, TPs displayed fine arborization and became more corrugated. TCs acquired signs of exaggerated secretory activities in the spring; TPs became expanded and packed with secretory vesicles. Thus, we conclude that, hormonal alterations during the reproductive cycle impact the morphology and secretory behavior of TCs.

## Introduction

Telocytes (TCs) represent a distinct type^1–4^ of stromal cells. They establish a cellular communication system and play a central role in the functional regulation of different types of cells and structures^5^. TCs have unique morphological features that distinguish them from other stromal cells. TCs possess cell prolongations; telopodes (TPs) which extend from the cell body. TPs form an interstitial labyrinthine network to allow cellular communication. TPs have thin segments or podomeres and interval expansions or podoms which are rich in mitochondria, endoplasmic reticulum, and caveolae^6^.

Several broad communication functions have been described for TCs. TCs transmit nerve impulses to smooth muscle cells^7–10^ and are involved in mechanoreception^11^. TCs also have excitatory and inhibitory neurotransmitter receptors^12^. The role of these cells in organ regeneration has been studied in several organs, including the heart, lung, skeletal muscle, skin, meninges and choroid plexus, eye, liver, uterus, and urinary system^13^. Based on gene expression analyses, various functions have been suggested for TCs such as cellular signalling^14,15^, cell expansion and movement^14^, tissue homoeostasis, remodelling^15^ and repair^13^, embryogenesis^16^, morphogenesis^17^, angiogenesis^15^, suppression of oxidative stress and cellular ageing^18^, and protection against inflammation and oncogenesis^19^.

Cellular connections are prominent feature of TCs that provide functional support to other cells. Two modes of communication are described for TCs; cell contact or through paracrine signaling. Cell contact is classified according to cell type into homocellular or heterocellular contact. TCs exhibit various forms of heterocellular contacts, including minute junctions such as point contacts, nanocontacts, planar contacts and cell contacts with an intermembrane distance, allowing macromolecules to interact^20^. The types of cell contacts in TCs including direct apposition of the cell membranes of adjacent TCs, adherence (puncta adherents minima, processes adherents, and manubria adherents), and gap junction. Gap junctions play a significant role in signal transduction between cells^20,21^. The paracrine functions of TCs depend on molecular transfer through extracellular vesicles; exosomes, ectosomes and multivesicular vesicles^6,22,23^.

Microenvironment- dependent changes in TCs behavior have been previously described; Hormonal administration affects the morphology and activities of TCs^24^. In the present study, we investigated TCs changes in response to hormonal changes in the genital organs in one of the seasonal breeder animals (camel) during the active and inactive reproductive seasons. We used samples of the efferent ductules for the one-humped camel obtained in two different seasons, summer and spring. Efferent ductules are important for sperm transportation and fluid reabsorption^25^. Camel breeding activity reaches maximum levels during the active or rutting period; in the winter and spring seasons while the breeding activity declines during the inactive or non-rutting period; summer and autumn^26,27^. The main goal of the present investigation was to recognize TCs in the efferent ductules, their distribution, identify camel TCs characteristics, their relations to other cells, and explore the activity of TCs during inactive and active seasons of the reproductive cycle.

## Materials and Methods

The present study was performed on efferent ductules collected from 10 clinically healthy mature camels (Camelus dromedarius) during the spring season (March-April) and during the summer season, which represents the inactive period (July-August). The materials were collected from the Bani-Adie slaughter house in Assiut-Egypt.

Efferent ductules were dissected and were fixed using (a) Bouin’s solution for light-microscopic examination and (b) Karnovsky fixative (10 mL of 25% paraformaldehyde, 10 mL of 50% glutaraldehyde, 50 mL phosphate buffer, and 30 mL distilled water, DW) for semithin sections and electron microscopy.

### Tissue processing

Samples fixed in Bouin’s solution were extensively washed in 70% ethanol (3 times for 24 h) to remove the fixative prior to subsequent tissue processing steps for paraffin block preparation; Fixed samples were dehydrated in ascending grades of alcohols at 70%, 80%, 90% and 100% for 90 min at each concentration. The samples were cleared using methyl benzoate. Dehydrated samples were then impregnated and embedded in Paraplast (Sigma Aldrich, USA). Serial sections of 3–5 μm were cut using a Richert Leica RM 2125 Microtome, Germany and mounted on glass slides. Sections were kept in an incubator at 40 °C.

### Acridine Orange (Fluorescent stain)

The procedure was according to that of Hoff, *et al*.^28^ with modification. Stock solution: 50 mg acridine orange is dissolved in 10 ml of distilled water and stored in the refrigerator, (0.5% AO. Staining solution: 1 ml of AO stock solution and 0.5 ml of glacial acetic acid were added to 50 ml of distilled water). The pH of the staining solution was approximately 3 and the AO concentration was 0.01%.

The staining procedure: 5-μm paraffin sections were dewaxed (2 times for 30 min) and rehydrated in a descending series of ethanol (100, 95, and 70%) and DW. Dried sections of glass slides were fixed with methanol and dried in a trough with AO staining solution (0.01 per cent). After 2 minutes of staining, the slides are washed gently with DW, dried and then analyzed using a Leitz DM 2500 microscope with the external fluorescent unit Leica EL 6000.

### Semithin sections

Specimens from efferent ductules were used for semithin sections. Small pieces 2.0–3.0 mm in length were fixed in Karnovsky fixative^29^ at 4 °C overnight. Semithin sections (1 μm) were cut using an ultramicrotome Ultracut E (Reichert-Leica, Germany) and stained with toluidine blue^30^.

### Immunohistochemistry

Different antigens (Table 1) were identified. The antigens were detected using either polyclonal or monoclonal antibodies using the avidin-biotin complex (ABC) technique^31^ according to the following protocol: paraffin-embedded tissue sections (5 μm) were dewaxed, rehydrated, and rinsed 3 times in PBS (pH 7.4) for 5 min. Endogenous peroxidase was inhibited by soaking the sections in 1% H_2_O_2_ for 10 min at room temperature, followed by intense washing under running tap water for an additional 10 min. For antigen retrieval, the slides were heated in water bath (for 20 min) in citrate buffer (pH 6.0) using a water bath followed by cooling for 20 min (Table 1). The sections were then rinsed 3 times with PBS (pH 7.4) for 5 min. The sections were covered with DAKO protein block serum-free solution (DAKO, Hamburg, Germany) for 10 min at room temperature to minimize nonspecific antibody binding (not exceeding 10 min to avoid a reduction in the desired level of staining). Then, the sections were incubated with the primary antibodies overnight at 4 °C or 1 h at room temperature. The sources, dilutions, and time of incubation of each antibody are shown in Table 1. The slides were washed with PBS (pH 7.4; 3.5 min), followed by incubation with a biotinylated secondary antibody (Table 1) for 30 min at room temperature. Thereafter, the slides were rinsed in PBS (pH 7.4; 3 5 min) followed by incubation with streptavidin-biotin-horseradish peroxidase complex (ABC solution; DAKO) for 30 min at room temperature.

**Table 1:** Identity, sources, and working dilution of antibodies used in immunohistochemical studies.

Target	Primary antibody supplier	Origin (catalog no.)	Dilution	Incubation	Ag retrieval	Biotinylated secondary antibody
Oestrogen receptor (EsR1)	Thermo fisher scientific^a^	Rabbit (PC; RM9101-S0)	1:200	1 h at RT	Microwave^a^	Goat anti rabbit IgG^a^
Progesterone receptor (PR)	Immunotech^b^	Mouse (MC; PR10A90	1:50	Overnight	Microwave^a^	Rabbit anti mouse IgG^a^
CD 34	Thermo fisher Scientific^a^	Rabbit (PC; PA1-39456)	1:200	Overnight	Microwave^a^	Goat anti rabbit IgG^a^
VEGF	Thermo fisher Scientific^a^	Mouse (MC; MA1-16629	1:200	Overnight	Microwave^a^	Rabbit anti mouse IgG^a^

PC = polyclonal; MC = monoclonal; RT = room temperature. a: microwave heating in citrate buffer (pH 6.0), 3 × 10 min.

^a^From ThermoFisher Scientific/Lab Vision, Fremont, CA, USA. ^b^Immunotec, Oxford, UK.

### Immunofluorescence

Paraffin sections were deparaffinized, hydrated with distilled water, followed by washing with 1x PBS. Afterwards, antigen retrieval (to decrease the masking of antigen epitopes because of tissue fixation with PFA) was carried out in 0.1 M sodium citrate buffer solution (pH = 6) for 4 min using a microwave (600 Watt). The sections were incubated at room temperature for 2 h with blocking solution (PBS containing 5% normal donkey serum (ABD Serotec), 1% bovine serum albumin (Roth) and 0.3% Triton X-100 (Fluka Bio Chemika) to minimize non-specific labelling and increase the permeability to the efferent ductule tissue. Sections were incubated 24 h at 4 °C with primary antibody against vimentin (rabbit monoclonal 1:100, Abcam) and androgen receptor marker (mouse monoclonal AR diluted in 1:200, Santa Cruz). To visualize the primary antibody the sections were first washed with PBS (3 × 10 min each), followed by incubation 2hrs (in dark) with the same blocking solution containing the secondary antibody conjugated to Alexa-594 (donkey anti-mouse 1:300, Cell Signaling Technology, Germany). Following a 1x PBS wash, sections were incubated for 10 min with DAPI (4′,6-diamidine-2′- phenylindole dihydrochloride, 1:10000, Roche, Germany) to visualize the nuclei, and FITC-coupled Tomatolectin (TL; 1:200, Sigma-Aldrich, Germany) to visualize the lectins (carbohydrate-binding proteins). The sections were washed again with PBS (3 × 10 min each). Finally, the sections were cover slipped with fluoro mount mounting medium (Dako, S3023), and were kept in the dark to preserve fluorescence until examination with an AxioImager M2 fluorescent microscope (Zeiss) and pictures captured with AxioCam HRc camera.

### Scanning electron microscopy (SEM)

Representative specimens from the efferent ductules were washed several times in normal saline and then fixed in a mixture of 2.5% paraformaldehyde and 5% glutaraldehyde in 0.1 M sodium phosphate buffer, pH 7.3, at 4 °C for 24 h. Thereafter the specimens were washed 4 times for 5 min in the fixation buffer and post fixed in 1% osmic acid in 0.1 M sodium phosphate buffer for additional 2 h at room temperature, followed by washing 4 times with 0.1 M sodium phosphate buffer for 15 min. The samples were dehydrated using different concentrations of alcohol; 50, 70, and 90% for 30 min at each concentration and 100% for 2 days (several changes) followed by isoamyl acetate for 2 days. The dehydrated samples were subjected to critical-point drying with a Polaron apparatus. Finally, the samples were coated with gold using JEOL- 1100 E ion sputtering device and observed with a JEOL scanning electron microscope (JSM 5400 LV) at 10 KV.

### Transmission electron microscopy (TEM)

Ultrathin sections were obtained by a Reichert ultra-microtome. The sections (70 nm) were stained with uranyl acetate and lead citrate and examined by a JEOL100CX II transmission electron microscope in the electron microscopy unit of Assiut University

### Color images

Scanned and transmission images were colored using the Photo Filter 6.3.2 program. To increase the visual contrast between several structures on the same electron micrograph.

### CMEIAS color segmentation: (for the supplementary images)

Negative images were performed using CMEIAS Color Segmentation, an improved computing technology used to process color images by segmenting foreground object of interest from the background^32^. This has been done by the following steps: open image with CMEIAS Color Segmentation, then select “Process” from the menu items, and subsequently choose “Negative image^33,34^”.

## Results

The present study aimed to identify TCs in the camel efferent ductules and investigate the morphological changes of TCs and their secretory behavior in response to seasonal variation.

### General organization of TCs

**Figure 1:**
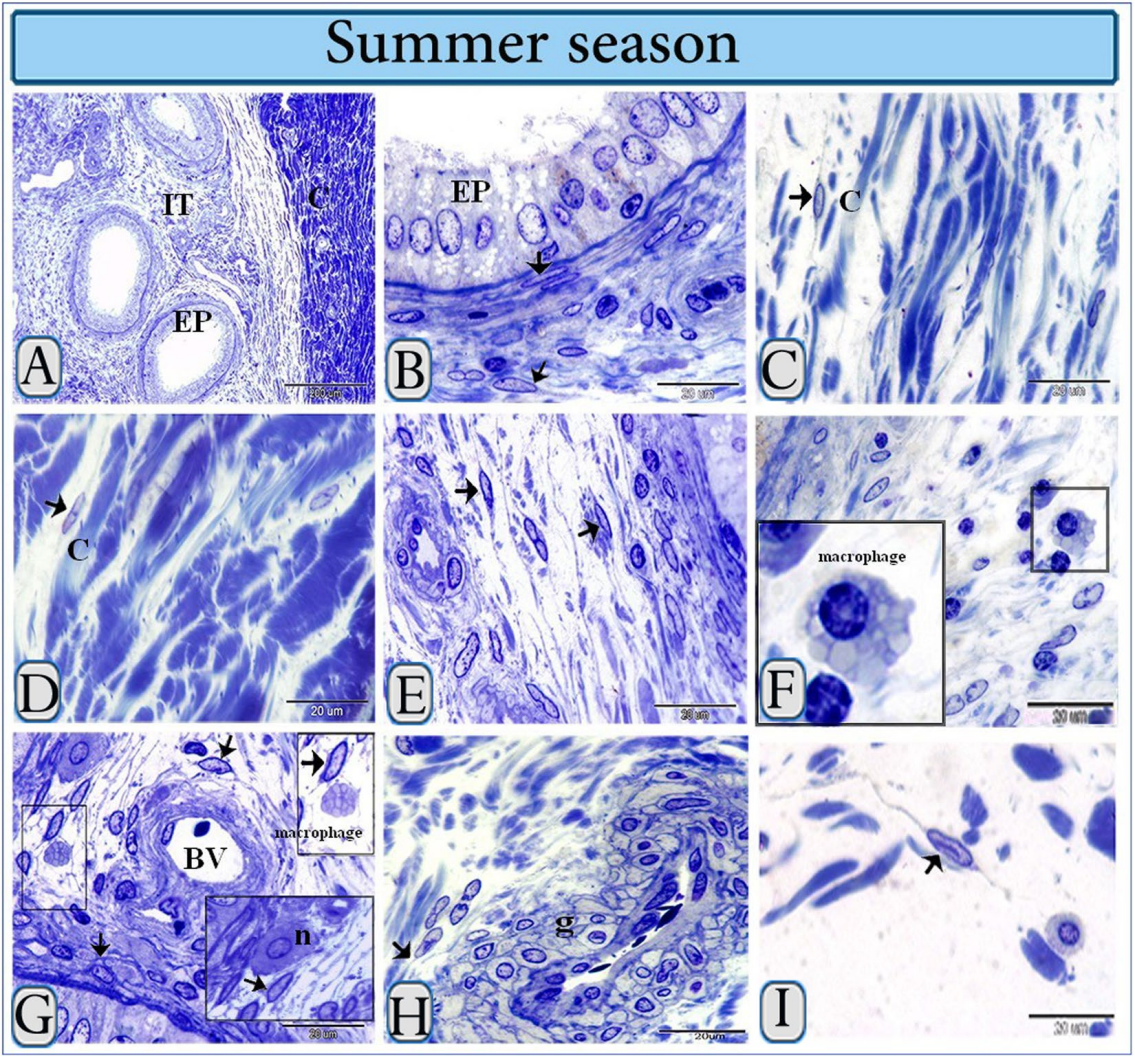
General organization of TCs in camel efferent ductules during the summer season. Semithin sections stained with methylene blue. (**A**) The general architecture of the efferent ductule of a camel. Note the epithelium (EP), interstitial connective tissue (IT) and capsule (C). (**B**) Sub-epithelial TCs (arrow). (**C**,**D)** TCs (arrows) distributed between collagen bundles of the capsule (C). (**E**) TCs in the interstitial stroma (arrows), (**F**) (framed area): Inactive macrophages were rich in secretory vesicles and lysosomes and were devoid of phagocytic inclusions. (**G**) TCs (framed areas) establish a connection with macrophage and neuroendocrine cells (n). Note: The arrow indicates TCs around a blood vessel. (**H**) TCs (arrow) around the glomus (g). (**I**) TCs in the interstisium, the nucleus contained inclusion bodies in the nucleus.

**Figure 2:**
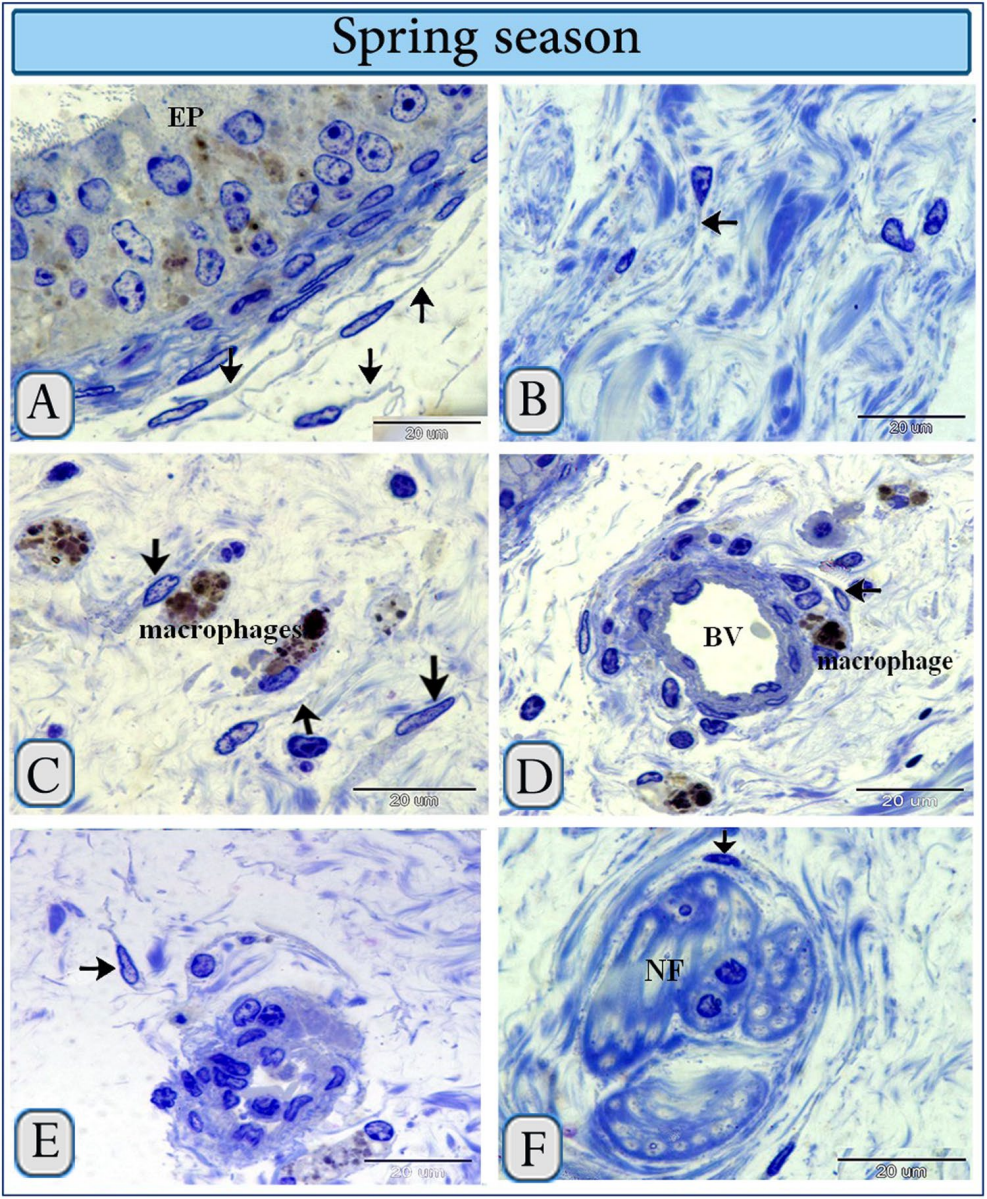
General organization of TCs in camel efferent ductules during the spring season. Semithin sections stained with methylene blue. (**A**) Sub-epithelial TCs (arrows), Note the epithelium (EP). (**B**) Interstitial TCs (arrow) located between collagen fibers. (**C**) TCs (arrow) in the interstitial stroma adjacent to macrophage that had prominent phagocytic inclusions. (**D**,**E**) TCs (arrow) around a blood vessel. (**F**) TCs (arrow) around nerve fibers.

TCs were identified in camel efferent ductules and, these cells were composed of the a cell body and TPs. TCs in camel efferent ductules were organized under the epithelium (Figs 1B and 2A), in the capsule (Fig. 1C,D) and interstitial connective tissue (Figs 1E and 2B,C). TCs were associated with inactive (Fig. 1F,G) and active (Fig. 2C,D) macrophages. TCs were observed around blood vessels (Figs 1G and 2D), neuroendocrine cells (Fig. 1G), the glomus (Fig. 1H) and nerve fibers (Fig. 2F).

### Identification and characterization of camel TCs

**Figure 3:**
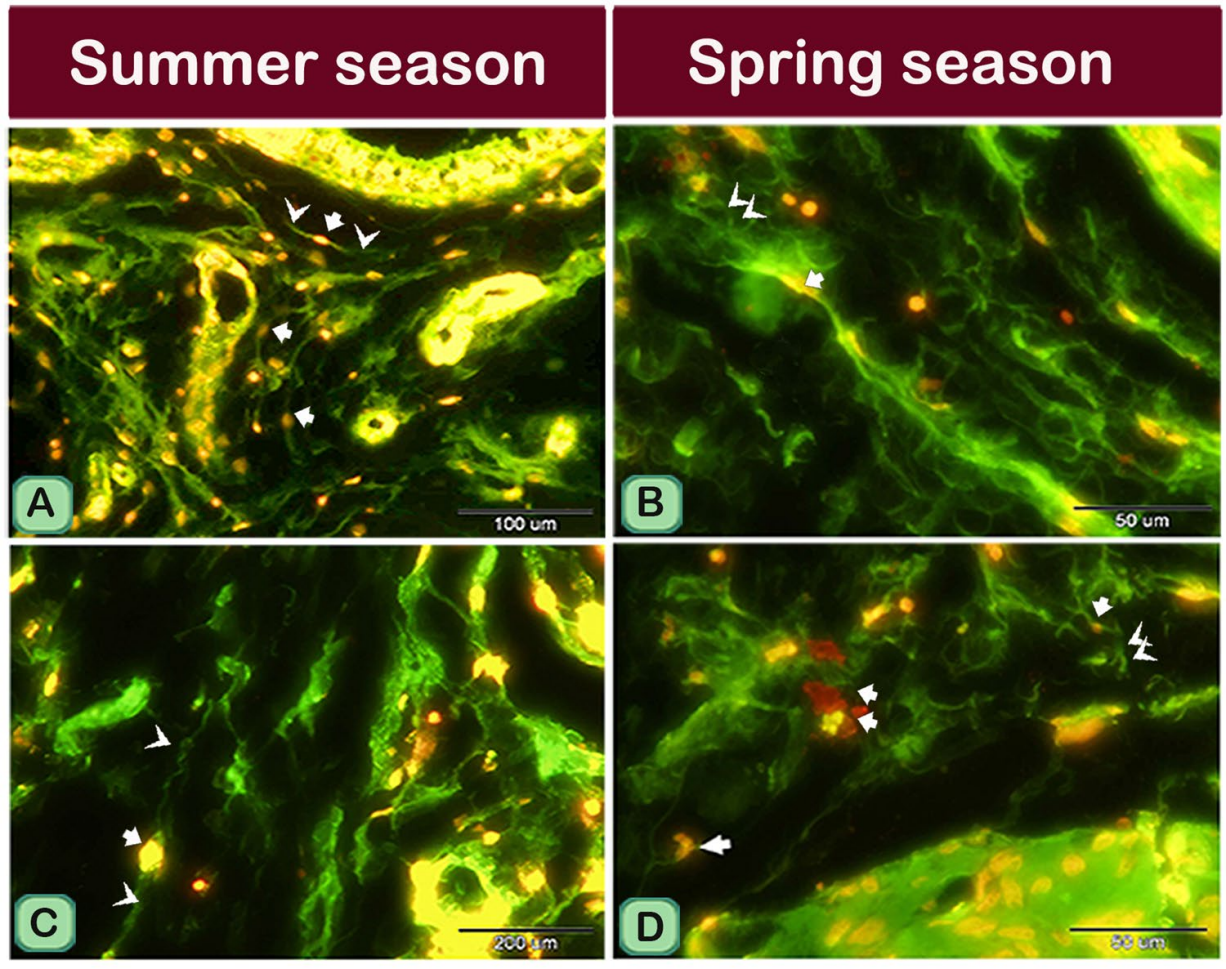
TCs in camel efferent ductules during the spring and summer seasons stained by acridine. TCs in the summer (**A**,**C**) and spring (**B**,**D**) seasons were identified by acridine. A were detected under the epithelium (EP) and interstitial stroma (**B**–**D**). Arrows indicate the cell bodies of the TCs, arrowheads refer to TPs. Note: that TPs gave rise to fine arborization (double arrowheads).

The typical morphological features of TCs were identified for the first time using “Acridine Orange staining technique”. As observed in Fig. 3 TCs were distributed under the epithelium and in the interstitial stroma in the summer and spring seasons (Fig. 3A–D).

### Immunohistochemical and immunofluorescence features of TCs

**Figure 4:**
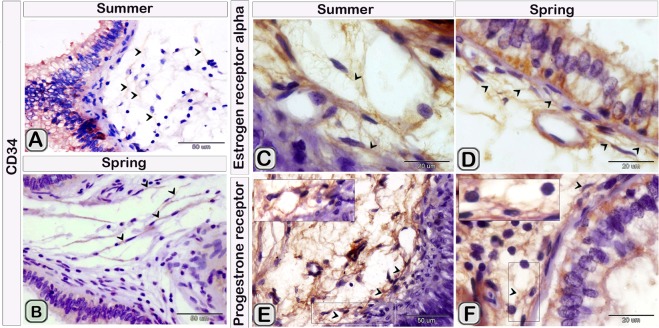
TCs immunoreactivity for CD34, ESR1 and PR in camel efferent ductules during the spring and summer seasons. Paraffin sections of the efferent ductule of camel in the summer and spring that underwent immunohistochemical staining for CD34 (**A**,**B**), ESR1 (**C**,**D**) and PR (**E**,**F**). Note that TCs showed a positive reaction for CD34 (**A**,**B**) in both summer and spring. TCs exhibited strong immunoreactivity for ESR1, complex estrogen receptors positive network in the spring (**D**) than in the summer (**C**). TCs showed a weak affinity for the PR immunostaining in summer samples (**E**) but a strong affinity for the PR immunostaining in the spring samples (**F**).

**Figure 5:**
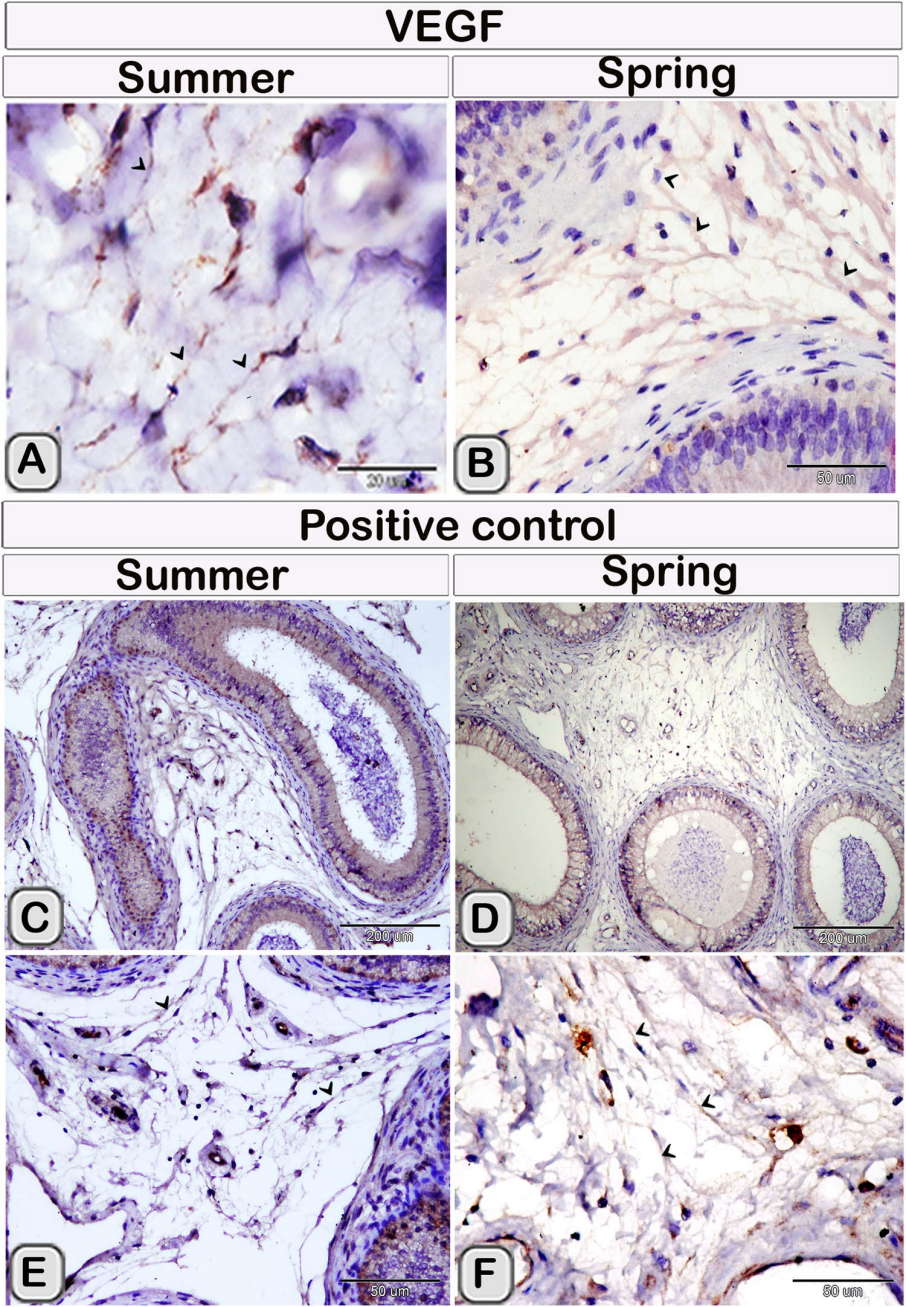
TCs immunoreactivity for VEGF in camel efferent ductules during the spring and summer seasons. Paraffin sections of camel efferent ductule in the summer and spring that underwent immunohistochemical staining for VEGF. TCs expressed strong immunoreactivity for VEGF in summer (**A**) than in the spring (**B**). Note that, (**C**–**F**) pictures showed positive control for camel epididymis in the respective seasons displayed stronger reaction in summer than in the spring.

TCs identification were confirmed by using CD34 immunohistochemical staining, their expression was present in cell bodies and TPs (Fig. 4A,B). TCs had strong immunoreactivity for oestrogen receptors (ESR_1_). Notably, ESR1 positive network was more complex in the spring than in the summer (Fig. 4C,D). TCs showed a weak affinity for progesterone receptors (PR) immunostaining in the summer samples. In contrast, it exhibited a strong affinity for PR immunostaining in spring samples (Fig. 4E,F). TCs cell body and TPs also were expressed VEGF. Moreover, the expression of VEGF was stronger in summer than spring season (Fig. 5A,B).

**Figure 6:**
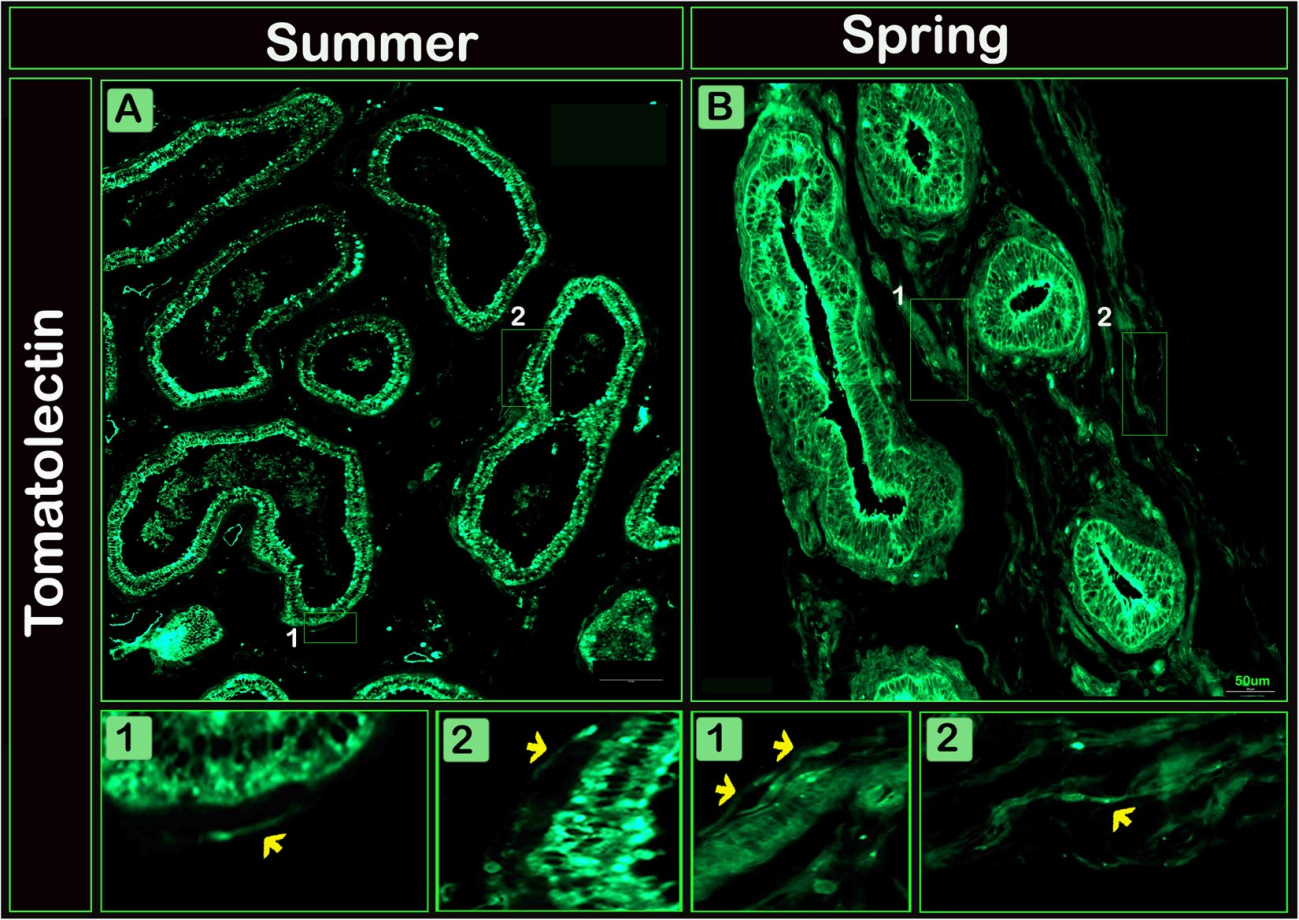
Immunofluorescence for Tomatolectin in TCs of the efferent ductules of camels during the spring and summer seasons. Paraffin sections of the efferent ductules in summer (**A**) and spring (**B**) TCs (arrows) showed positive immunoreactivity (green) for Tomatolectin.

**Figure 7:**
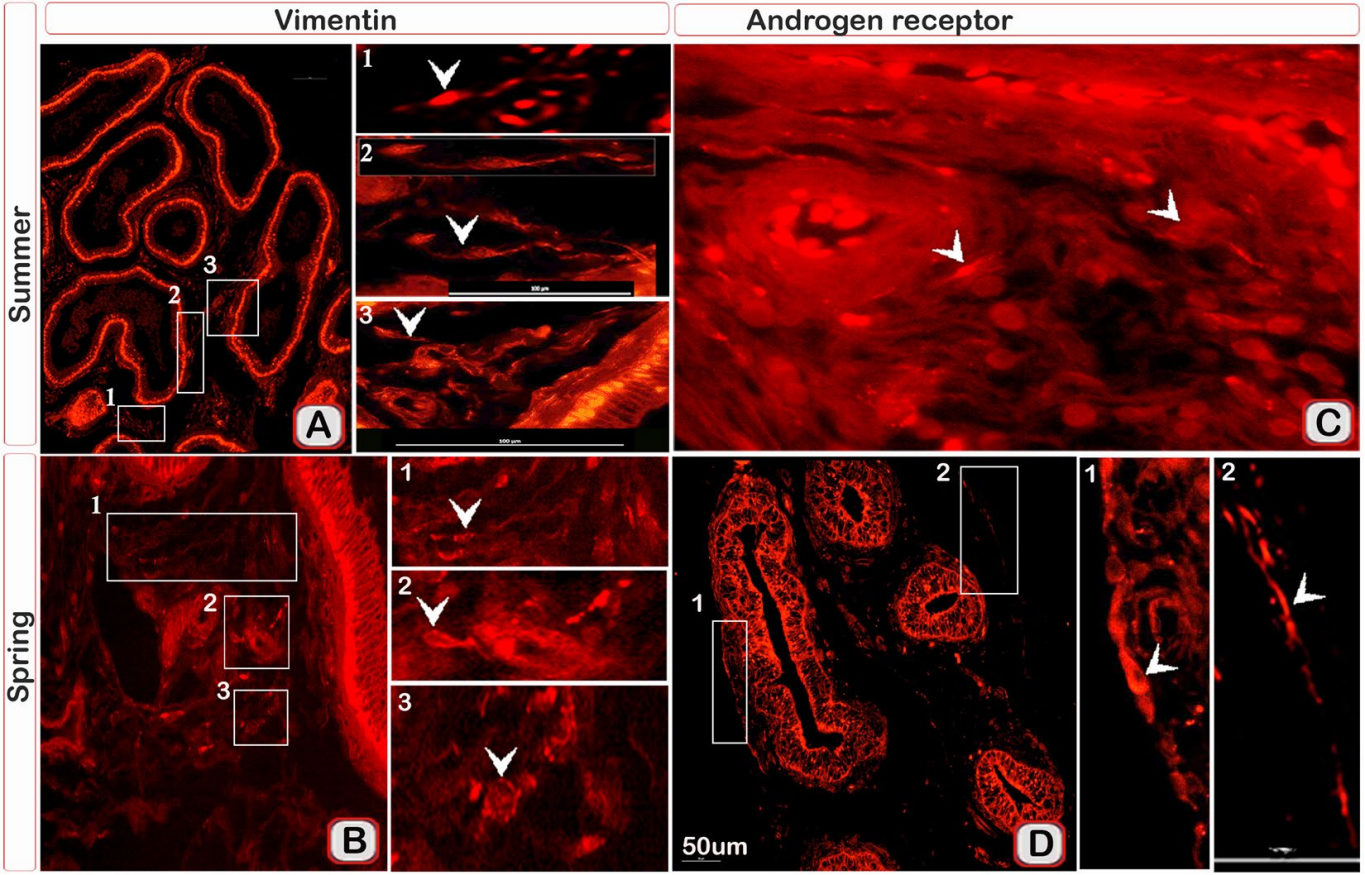
Immunofluorescence for Vimentin and AR in TCs of the camel efferent ductules during the spring and summer seasons. Paraffin sections of the efferent ductules in summer (**A**,**C**) and spring (**B**,**D**) showed positive immunoreactivity (red) for Vimentin and AR in TCs (arrow heads).

We used Tomato lectin immunostaining for more identification of TCs features, TCs showed positive reaction for Tomato lectin in both seasons (Fig. 6). In addition, TCs showed a strong positive reaction for both vimentin (Fig. 7A,B) and AR (Fig. 7C,D) immunostaining.

### Scanning electron microscopy

**Figure 8:**
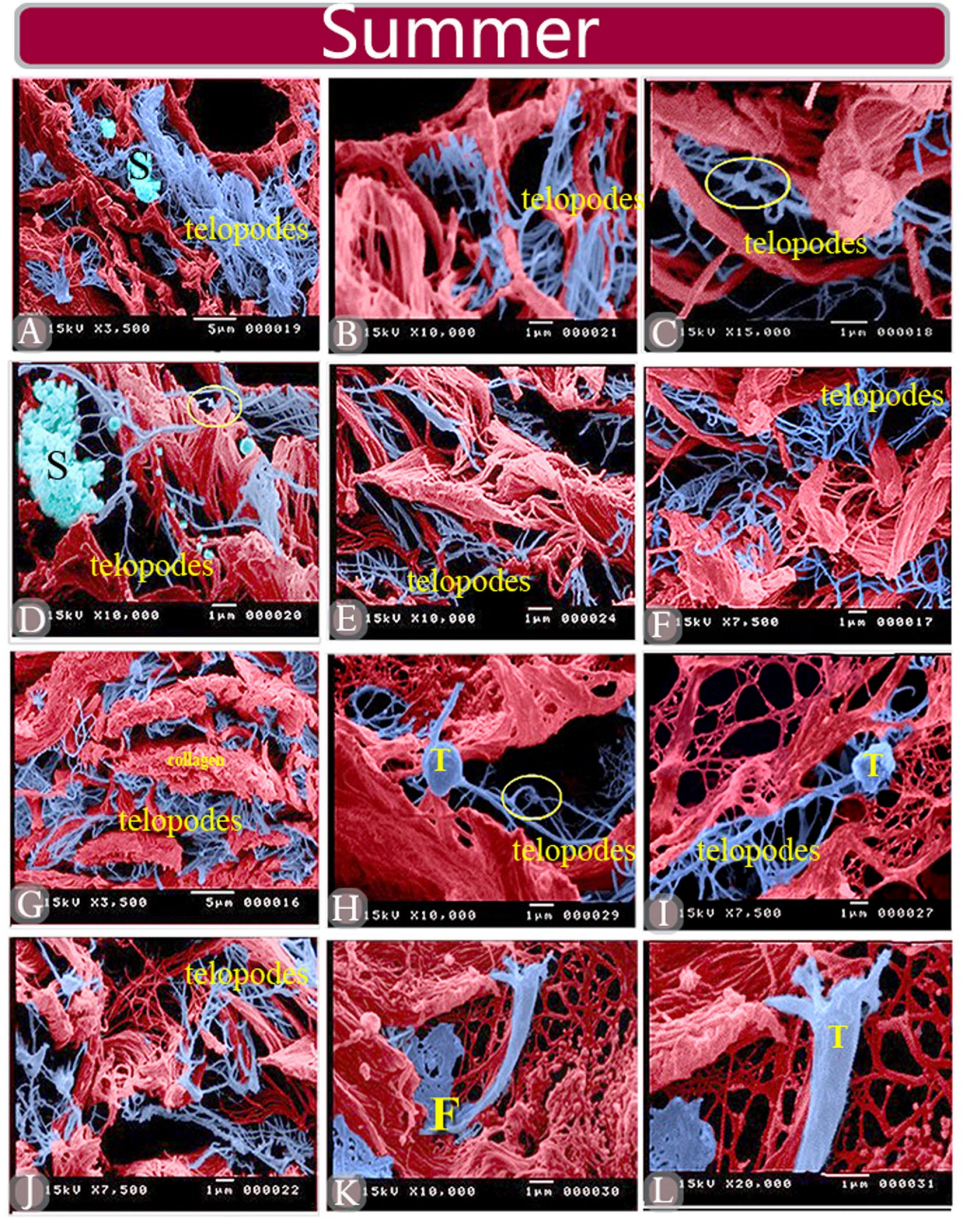
Scanned samples of camel efferent ductules in summer season. (**A**–**C**). The blue color refers to a 3D TPs network in the interstitial stroma of the efferent ductules. Note TCs secretion (S) Podoms are enclosed by yellow circles. (**D**–**L**) TCs (T) located in the interstitial stroma. (**I**) TPs formed a network, which was distributed between collagen bundles of the capsule. (**H**) TCs (T) were spherical and triangular in (**K**,**L**) in shape. Note the fenestrated membrane (**F**) extending from the TPs (**K**).

**Figure 9:**
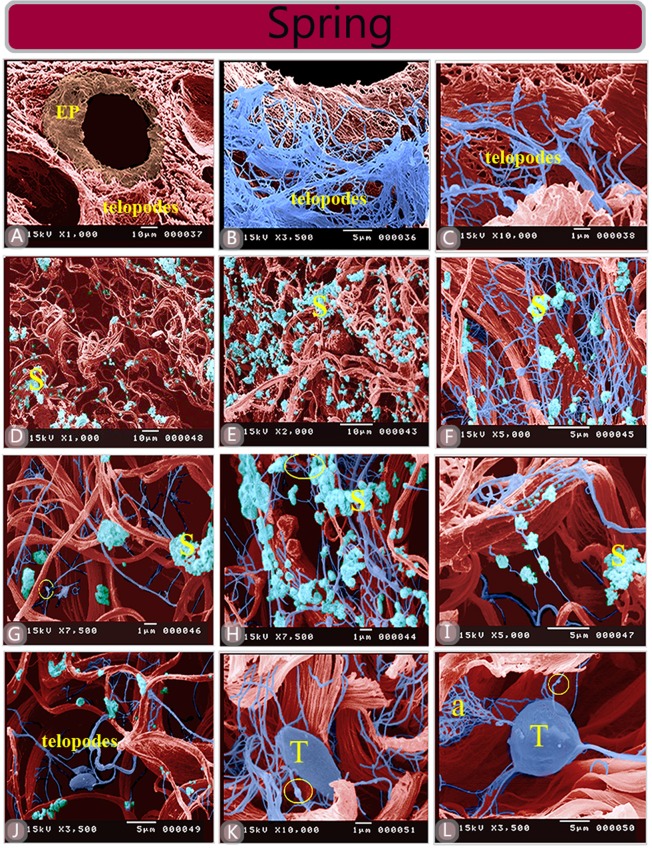
Scanned samples of camel efferent ductules in the spring season. (**A**) General view of the efferent ductules. Note, the epithelium (EP). (**B**,**C**) Higher magnification of panel A. (**D**–**J**) Abundant secretory vesicles of TCs (S) that shed form TCs. (**F**–**J**) 3D TPs network. (**K**) Large oval-shaped telocyte (T). Note, that the yellow circle refers to the podom. (**L**) Spherical-shaped TCs (T). Note, TPs may exhibit fine.

TCs were also identified by SEM in the summer and spring. TCs in summer had ramifying TPs (Fig. 8A–E). Few secretory vesicles were shed from TCs during the summer (Fig. 8A,D). TPs continued with an expanded fenestrated sheath or fenestrated membrane (Fig. 8K). TCs were spherical (Fig. 8H) triangular (Fig. 8K,L). TCs during the spring season were also examined by SEM (Fig. 9A–K). The most prominent change in TCs during the spring was that these cells exhibited an exaggerated secretory activity. Many secretory masses were shed from TCs (Fig. 9D–J). Some TCs became enlarged in size (Fig. 9K,L). TPs exhibited fine arborization (Fig. 9L).

### Ultrastructural differences of camel TCs during the spring and summer seasons

**Figure 10:**
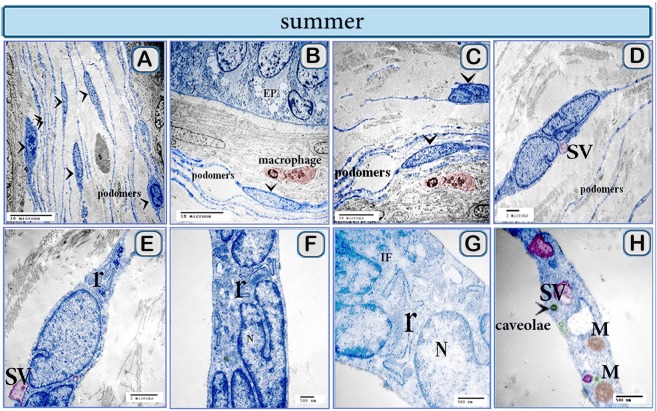
Colored ultra-thin sections of camel efferent ductules during the summer season. (**A**–**C**) Abundant TCs were identified in the interstitial stroma. TCs had cell bodies (arrowheads) Podomeres were ramified and formed a 3D network, note: epithelium (EP), macrophage. (**D**–**H**) TCs had RER (r), secretory vesicles (SV), mitochondria (m), intermediate filament (IF) and caveolae. Note that TCs nucleus may be indented (N) in “C-G”.

**Figure 11:**
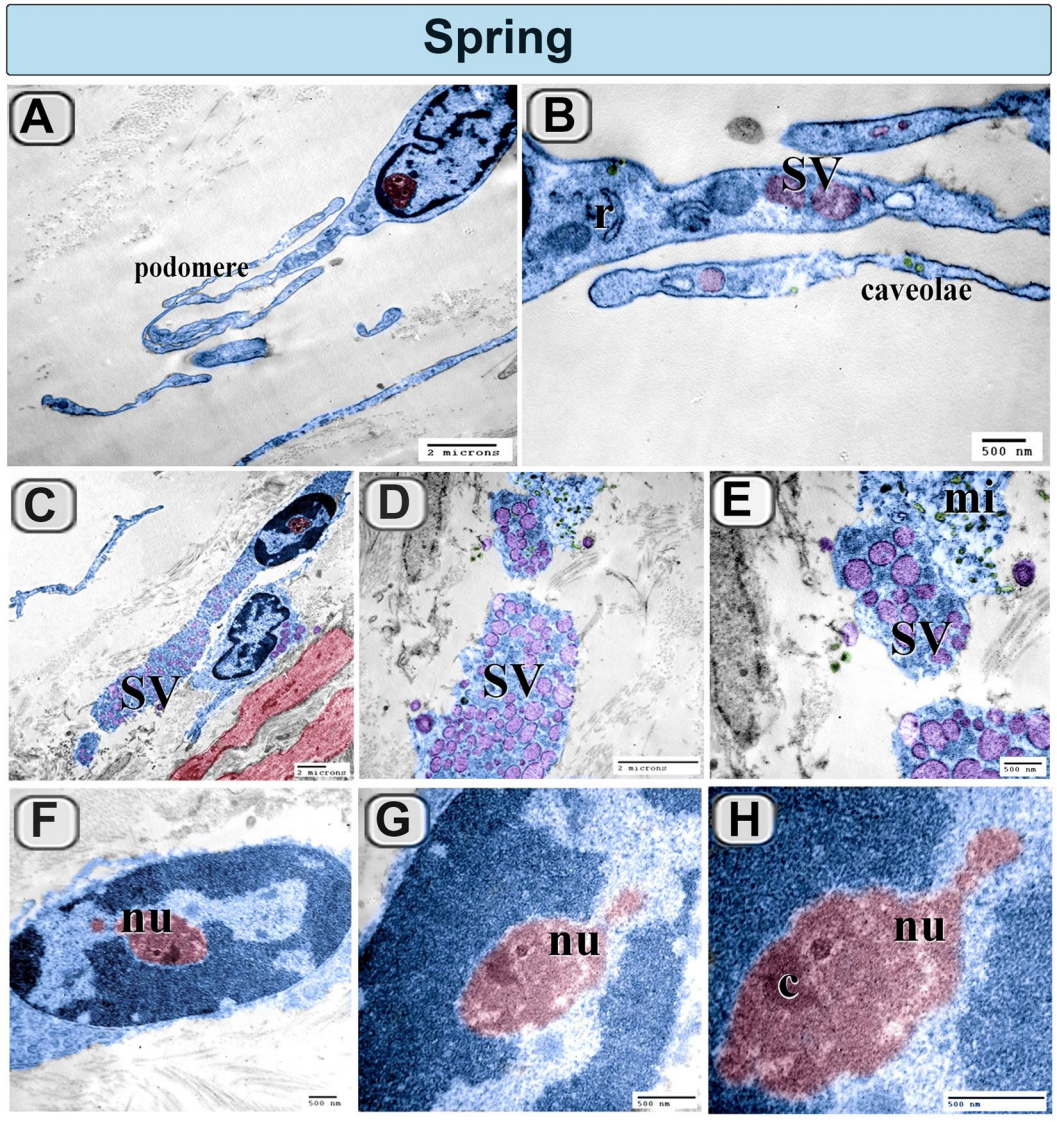
Colored ultra-thin sections of camel efferent ductules during the Spring season. (**A**,**B**) TCs (blue colored) had corrugated podomeres. (**B**) TCs (Blue colored) have RER (r), secretory vesicles (SV) and caveolae. (**C**–**E**) Some TCs had dilated TPs and packed with secretory vesicles (SV). Note, the microvesicles (mi), SMF (red colored). (**F**–**H**) the nucleus of TCs exhibited prominent nucleolus (nu) which contained a well-defined intranucleolar chromatin (c).

**Figure 12:**
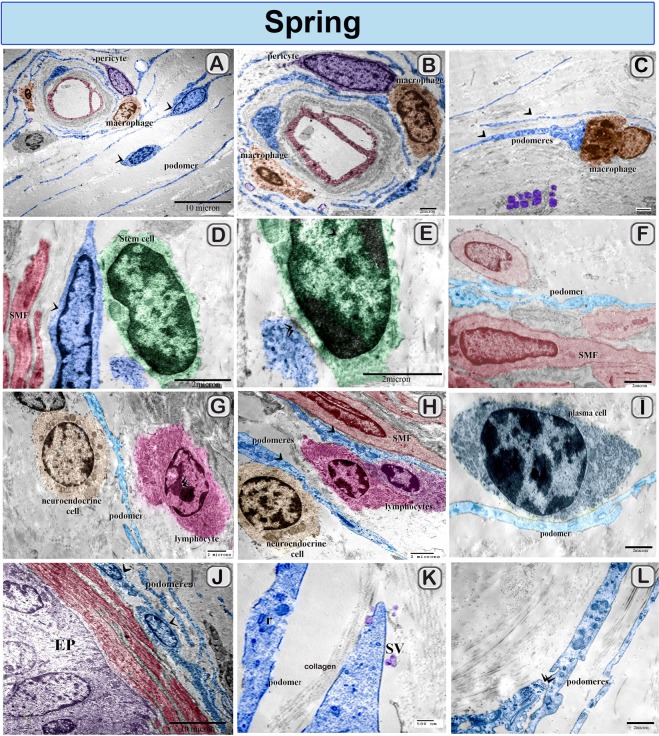
Colored ultra-thin sections of camel efferent ductules during the Spring season showing contact with different types of cells (**A**,**B**) Podomeres of TCs (arrowheads) were observed around the blood vessels. Note pericytes (violet colored) and macrophages (brown colored). (**B**,**C**) TCs connect with macrophages and pericyte. Note. macrophage rich in lysosomes in "C". (**D**,**E**) TCs established direct contact with stem cell. Note the electron dense area (double arrowheads). Smooth muscle fibers (red colored). Stem cell (green color) was identified by its high nuclear/cytoplasmic ratio and had mitochondria. (**F**) Podomere connected with smooth muscle fiber (red colored). (**G**,**H**) Podomeres established contact with neuroendocrine cells and lymphocytes. (**I**) Podomere formed contacts with plasma cell. (**J**) homocellular contact between cell bodies (arrow heads) of two adjacent TCs, note: epithelium (EP). smooth muscle fibers (red colored). (**K**,**L**) podomere contained RER (r) and secretory vesicle (SV). Note homocellular contact (double arrowheads).

Morphological differences in TCs were observed by TEM in the summer and spring. In the summer TCs had small cell body; TPs showed distinct podomeres and TPs had delicate ramifications and formed a 3D network (Fig. 10A–C). The indented nucleus was a characteristic feature of camel TCs (Fig. 10D–F). They had rough endoplasmic reticulum (RER), secretory vesicles, caveolae, mitochondria and intermediate filaments (Fig. 10D–H). TCs underwent morphological alterations in the spring season. TPs had corrugated podomeres (Fig. 11A,B). Signs of high TCs secretory activities were observed; TPs became expanded and packed with secretory vesicles (Fig. 11C–E). TCs also shed microvesicles (Fig. 11E). TCs exhibited a prominent intranucleolar chromatin (Fig. 11F–H). TCs in spring showed contact with different types of cells including; TCs with pericytes and active macrophages that rich in lysosomes (Fig. 12A–C), stem cells (Fig. 12D,E), and heterocellular contact was established with smooth muscle cells (Fig. 12F,J), lymphocytes, neuroendocrine cells (Fig. 12G,H) and plasma cells (Fig. 12I), homocellular contact was observed (Fig. 12K,L).

**Figure 13:**
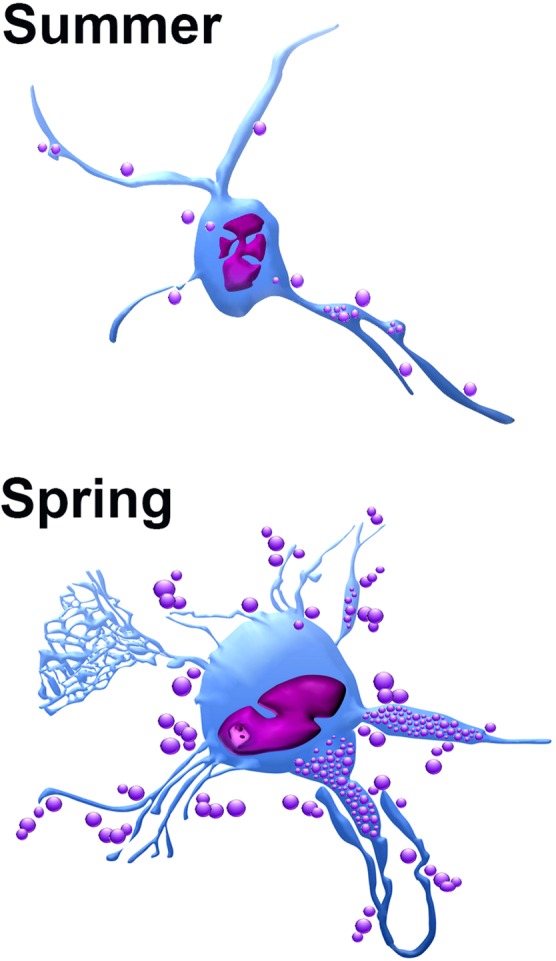
Illustration showed the morphological differences in TCs in the summer and spring. TCs in the summer had small cell body, TPs with distinct podomeres. The indented nucleus may be a characteristic feature of camel TCs. TCs undergo morphological alterations in the spring season. TPs may have corrugated podomeres. Signs of high TCs secretory activities were observed; TPs became expanded and packed with secretory vesicles. TCs also shed microvesicles.

Seasonal morphological changes of TCs in the summer and spring are summarized in (Fig. 13).

## Discussion

Reproduction have taken a great concern in the research field^35,36^. TCs in the genital tract influence the reproductive function. Thus, the present study provided evidence for the existence of TCs in the stroma of the camel efferent ductules and investigated their organization, distinct camel TCs characteristics, relations to other cells, and morphology and their possible role in reproduction during active and inactive seasons of the reproductive cycle.

TCs identification was based on morphological criteria, using different techniques; SEM, TEM, and immunophenotyping. TCs have been identified in the mammalian testis of human^37^, mouse^38^, rabbit^39^. Camel TCs possessed features identical to those of TCs in other mammalian species^40–43^. Most of TCs had indented nuclei; nuclear indentation may be a characteristic feature of camel TCs.

TCs are difficult to visualize using classical stains, but in the present study we visualized TCs for first time using the “Acridine Orange stain”. The AO staining technique is simple, sensitive and screening technique^44^, for detection of TCs this stain is easier and cheaper than immunofluorescent method. TCs had a cell body and multiple TPs which gave rise to delicate ramifications. We visualized both the morphological features of TCs and their secretion using AO. TCs arborization were identified in the spring and their secretion was detected in the extracellular matrix which stained orange to red color. AO is a cationic dye and reacts with vesicle-associated membrane proteins such as secretory vesicles as well as the membrane bounded acidic compartments, the lysosomes which exhibit low pH environment. AO have metachromatic properties that results in the concomitant emission of green and red fluorescence. AO stained the membrane bounded vesicles and give rise an orange to red reaction. Thus, AO is commonly used to detect the secretory vesicles and lysosomes. AO was used as an optical probe to quantify trans membrane pH gradients in the biological vesicles^45,46^.

TCs expressed strong immunoreactivity for Tomatolectin, Lectins are proteins or glycoproteins of non-immune origin^47^. This finding suggests that, TCs in the efferent ductules shared molecular features of sperm plasma membrane, lectins mostly expressed at the acrosome, post acrosomal region, and mitochondria associated with the middle piece. Alternations of lectin binding is associated with sperm maturation in the epididymis, during capacitation, and after the acrosome reaction suggesting a role of lectins sperm maturation^48^. Duct cells in urodele testis rich in lectin, the authors suggested that lectin may provide an adequate environment for sperm storage, conservation and maturation^49^. Moreover, lectins may involve in sperm-egg interactions^48^.

In the present study, TCs exhibited a distinctive organization in the efferent ductules. These cells were located under the epithelium, in the interstitial stroma and in the capsules between collagen bundles. A similar distribution was recognized in the glandular organ^40^. Whereas TCs distribution was different in the tubular organs, as these cells were organized in each layer^41^. That organization may suggest that TCs may be involved in transportation of spermatozoa and testicular fluid^50^. In the present study, TCs were embedded in the collagen fibers that may be play a role in the regeneration and repair of the interstitial tissue of the efferent ductules. Our findings are in line with those of previous studies that have reported that skin TCs are found near collagen and elastic fibers^51^.

As reported in the present study, TPs communicate with other TCs and other cells, including stem cells, macrophages, lymphocytes, plasma cells, neuroendocrine cells, and smooth muscle cells. These results suggested that TCs play a potential role in efferent ductules regeneration, endocrine regulation and immune responses. In the current study, TCs communications with macrophages supported a potential role for TCs in the indirect regulation of phagocytotic activities. TCs might be considered as an immune system regulator connecting the immune cells in the interstitium and providing functional support^52^. In the present work, TCs were observed in vicinity and in direct contact with macrophages. Macrophage which established direct contact with a TP exhibited signs of active phagocytic cells and became rich in lysosomes comparing with free macrophage. we suggested that TCs may regulate the phagocytic activities of the macrophages. Immunoregulatory and immunosurveillance functions have been suggested for TCs. They are implicated in regulation of the functional activities of the macrophages via mitochondrial signaling pathway^53,54^.

The communication between TCs and stem cells indicated a potential role for TCs in tissue regeneration. A similar conclusion was reported in the heart; after joining with stem cells, TCs are involved in the regeneration and repair of myocardial infarction^55^. TCs secret small or large molecules (proteins or RNAs) that influence stem cells by paracrine or juxtracrine mechanisms^56^. Moreover, TCs established contact with blood vessels, glomus, nerve fibers and the epithelium. Hence, TCs likely serve as transducing centers that provide cell singling via a TPs network to other cell types and structures^6^.

The present study described for the first-time changes in TCs behavior during the active and inactive breeding seasons in the efferent ductules of the camel. TCs exhibited moderate seasonal differences in their morphological features and activities. Some characteristics (morphology and density) of TCs change with some conditions^57^. These results may be attributable to hormonal variations between the two seasons. Gonadal activity is increased during the spring and declines during the summer in the camel^27^. Thus, TCs play an important role in male fertility. The impairment of oviduct TCs leads to infertility^58^.

TCs share immunological marker characteristics of undifferentiated stem cells, such as CD34 and vimentin. In the current study, all TCs in the efferent ductules of camel were CD34, VEGF and vimentin positive. Camel TCs express CD34 similar immunophenotype of TCs in other mammalian species^42^ as well as avian species^59^, reptiles^50^, amphibians^60^ and clitellates species^2^. CD34/PDGFR-α has been identified as a specific marker for TCs^61^. CD34 is a transmembrane phosphoglycoprotein that identified on hematopoietic stem and other progenitor cells including muscle satellite cells, corneal keratocytes, interstitial cells, epithelial progenitors, and vascular endothelial progenitors^62^. Vimentin positive TCs are also recognized in various tissues and organs such as uterine tube and uterus^63^, lung^18^, placenta^64^, mammary gland^65^, and heart^66^. Vimentin is one of the intermediate filaments provide structural support to maintain cellular integrity and protective function against stress^67^. Expression of vimentin is associated with normal development, cellular transformation, and growth in tissue culture^68^. Vimentin is expressed in a wide range of cells; fibroblasts, endothelial cells in blood vessels, epithelial cells, macrophages, cells of cartilage and bone, some vascular smooth muscle cells and melanocytes^68^. In addition, TCs exhibited stronger immunoreactivity for ESR1, PR and AR in the spring than in the summer. Steroids, particularly estrogen, progesterone and androgen mediated their actions through ESR, PR and AR^69,70^. Oestrogen plays a vital role in efferent ductule functions to reabsorb testicular fluid; thus, the lack of ESR1 may lead to a disturbance in the epithelial morphology and inhibit reabsorption of the testicular fluid^71^. However, progesterone affects male fertility and testosterone biosynthesis^72^. There is considerable evidence suggesting that, TCs are important targets for steroid hormones. TCs can function as ‘hormonal sensors’ in the human reproductive tract because they express progesterone and estrogen receptors^8^. Previous studies have reported the presence of ESR and PR on TCs of the myometrium^73^, fallopian tubes^41^, Seminal vesicles^40^. TCs in the fallopian and myometrium tissues may act as steroid sensors^74^. TCs may be involved in androgens secretion as reported before, they are connected to Leydig cells^50^.

TCs could play a potential role in the development of blood vessels and endothelium. Vascular endothelial growth factor (VEGF) is one of the signaling protein belongs to the platelet-derived growth factor (PDGF) family. VEGF promote angiogenesis^75^, maintain vascular integrity^76^ and regulate vascular permeability^77^. Hence VEGF is termed as the vascular permeability factor (VPF). VEGF increase the vascular permeability by the endothelial cells through increasing the fenestrations^78^. Both VEGF and PDGF Receptor-α are expressed by TCs^79,80^. In the current study, VEGF was also expressed by camel efferent ductule TCs. The seasonal variation of TCs VEGF immunoreactivity was observed. TCs in the inactive summer season express strong immunoreactivity for VEGF more than the active spring season. Diminishing expression of the VEGF in the spring may serve in the thermoregulation that required for spermatogenesis. VEGFA have a significant role in the regulation of vascular permeability as well as in spermiogenesis and the proliferation of spermatogonia^81,82^.

This result agree with earlier report^83^ demonstrating that VEGF expression in the testis is downregulated during the mating season in one of the seasonal breeder animals, roe deer. Moreover, Camels have adapted to the hypoxic condition of the desert during summer season, in turn the hypoxia activates hypoxia inducible factor 1 (HIF-1). Hypoxia-inducible factor 1 (HIF-1) is a transcription factor that involved in regulation oxygen consumption^84^. VEGF is one of the downstream gene of the HIF^85^. This may explain higher expression of the VEGF of TCs in the summer than the spring season.

Several ultrastructural changes were observed in TCs during the both seasons. TPs in summer had delicate ramifications whereas, TPs in spring may exhibit fine arborization and became more corrugated. TCs, to become able to connect with the other cells or structures through their TPs or by extracellular vesicles and play a potential role in the maintenance of the reproductive tissue homeostasis and renewal^86^. TCs acquired signs of exaggerated secretory activities in the spring. TPs became expanded and packed by secretory vesicles. As observed by SEM, a large number of secretory masses were shed from TCs. Some TCs became enlarged in size. It suggests that during the spring season, elevation of the reproductive activity, there was increased demand for extensive transmission of intercellular information that utilizes small molecules, exosomes^87^. These vesicles may contain various growth factors or cytokines, which are very important in regulating the microenvironmental factors^87^.

Intranucleolar chromatin was observed in TCs as well as lymphocytes. The structural organization of intranucleolar chromatin is associated with seasonal and hormonal variation. This finding suggests that the intranucleolar chromatin may act as a temporarily inactive gene that is activated when necessary to satisfy the functional demand in the reproductive cycle particularly the secretory function. Identification of the intranucleolar chromatin composition revealed that they represented as rDNA transcriptional units^88^.

In conclusion, seasonal differences in TCs morphology and behavior are likely regard to hormonal variations between the two seasons. Hormonal alternations during the reproductive cycle may influence the TCs morphology and secretory behavior. The results of the present study support the hypothesis that TCs are affected by microenvironmental changes. TCs gain additional short TPs in an environment characterized by oxidative stress. These cells also change their morphology and acquire a long and slender shape in N-acetyl cysteine cell culture medium^89^.

## Supplementary information

supplementary figures and legends
