# Author Correction: Morphological changes of telocytes in camel efferent ductules in response to seasonal variations during the reproductive cycle

**DOI:** 10.1038/s41598-020-59137-6

**Published:** 2020-02-06

**Authors:** Fatma M. Abdel-Maksoud, Hanan H. Abd-Elhafeez, Soha A. Soliman

**Affiliations:** 1 Department of Anatomy and Histology, Faculty of Veterinary Medicine, Assiut University, Assiut, Egypt; 2 Department of Histology, Faculty of Veterinary Medicine, South Valley University, Qena, Egypt

Correction to: *Scientific Reports* 10.1038/s41598-019-41143-y, published online 14 March 2019

The Legend for Figure 10 is incomplete, as it does not specify that the panels for Figure 10b and 10c overlap. As a result,

“(**A**–**C**) Abundant TCs were identified in the interstitial stroma. TCs had cell bodies (arrowheads) Podomeres were ramified and formed a 3D network, note: epithelium (EP), macrophage.”

should read:

“(**A**–**C**) Abundant TCs were identified in the interstitial stroma. TCs had cell bodies (arrowheads) Podomeres were ramified and formed a 3D network, note: epithelium (EP), macrophage. Panel 10b shows one telocyte cell under the epithelium (Ep), associated with a macrophage (selected square); Panel 10c provides a more focused image of the same area under the epithelium, showing another telocyte (arrow head) in addition to the previous one.”

