# Supplementary material for: Morphological changes of telocytes in camel efferent ductules in response to seasonal variations during the reproductive cycle
Source: Sci Rep. 2019 Mar 14;9:4507. doi: 10.1038/s41598-019-41143-y (PMC6418092; doi:10.1038/s41598-019-41143-y)
Supplement: Supplementary file 1 — supplementary figures and legends [file 41598_2019_41143_MOESM1_ESM.pdf]

# Morphological changes of telocytes in camel efferent ductules in response to seasonal variations during the reproductive cycle

Fatma M. Abdel-Maksoud [1], Hanan H. Abd-Elhafeez[1], Soha A. Soliman[2]
[1]Department of Anatomy and Histology, Faculty of Veterinary Medicine, Assiut University, Assiut, Egypt.
[2]Department of Histology, Faculty of Veterinary Medicine, South Valley University, Qena, Egypt.

***Abbreviated title:*** Telocytes in camel efferent ductules
***Key terms:*** Efferent ductule, telocyte, camel
***Number of supplementary figures:*** 3 figures
Corresponding author and person to whom reprint requests should be addressed:
Fatma M. abdel maksoud, PhD
Department of Anatomy and Histology
Assiut University, Assiut, Egypt, 71526.
Tel.: +101013792630
E-mail: fatma.abdelmaksoud@vet.au.edu.eg

**Supplementary figure 1:**

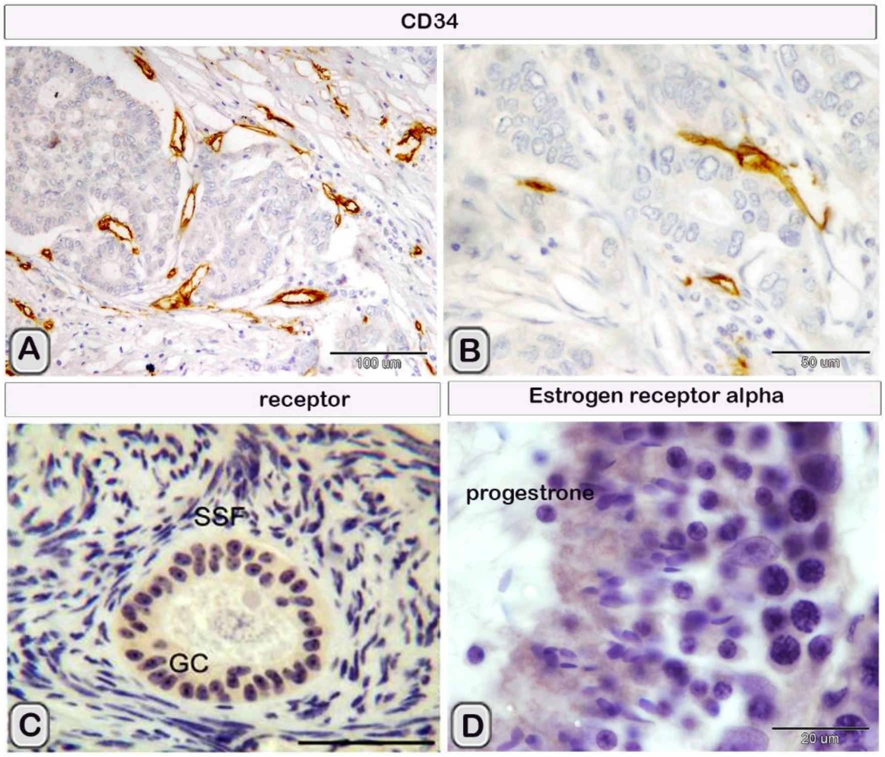


**Supplementary legend 1:**

Paraffin sections for gastrointestinal tumor (A, B), ovary cattle (C) and donkey testis (D) tissues underwent immunohistochemical staining as positive control used for positive control for CD34 (A, B), PR (C) and ESR1 (D).

**Supplementary figure 2:**

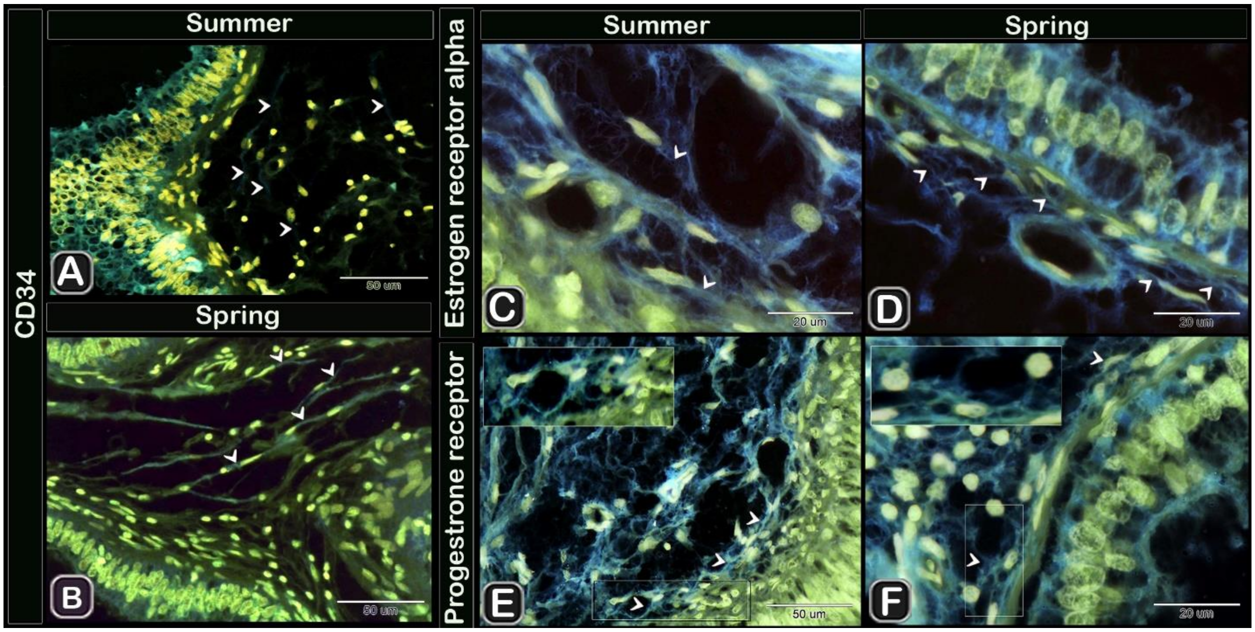


**Supplementary legend 2:**

Negative images for organization of CD34 (A, B), ESR1(C, D) and PR (E, F) positive TCs in camel efferent ductules during summer and spring seasons by using CMEIAS Color Segmentation.

**Supplementary figure 3:**

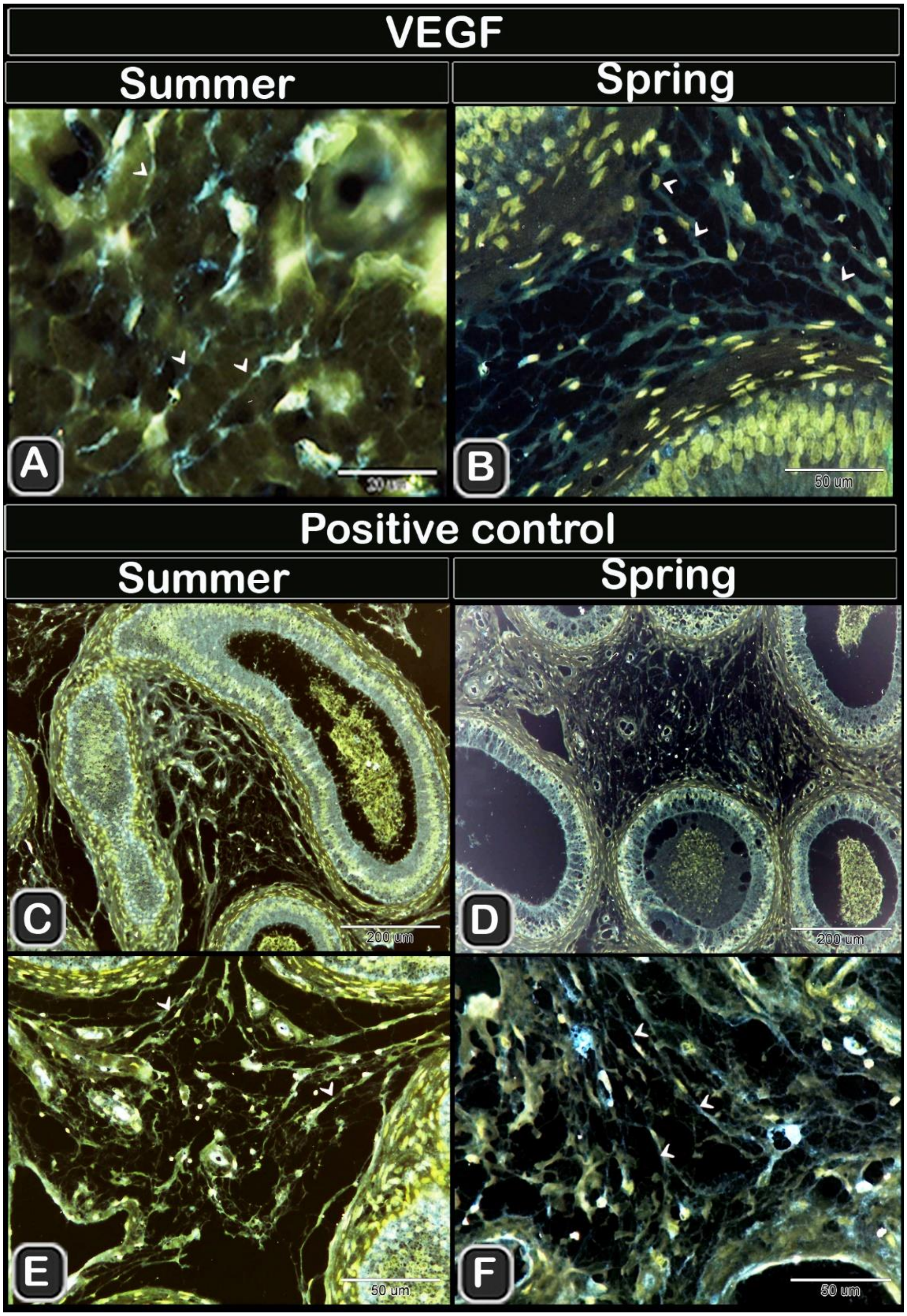


**Supplementary legend 3:**

Negative images for organization of VEGF positive TCs in camel efferent ductules (A, B) and camel epididymis (C-F) during summer and spring seasons by using CMEIAS Color Segmentation.
